# Lactic Acid Conversion to Acrylic Acid Over Fluoride-Substituted Hydroxyapatites

**DOI:** 10.3389/fchem.2020.00421

**Published:** 2020-05-13

**Authors:** Robert Wojcieszak, Thomas Bonnotte, Sébastien Paul, Benjamin Katryniok, Franck Dumeignil

**Affiliations:** Univ. Lille, CNRS, Centrale Lille, Univ. Artois, UMR 8181 - UCCS - Unité de Catalyse et Chimie du Solide, Lille, France

**Keywords:** hydroxyapatites, dehydration, fluorine, lactic acid, acrylic acid

## Abstract

One of the most interesting intermediates for the chemical industry is acrylic acid, which can be derived from lactic acid by catalytic dehydration in the gas phase. The realization of this reaction is complex due to a strong thermal activation leading to the formation of undesired by-products (acetaldehyde, propanoic acid…) as well as polymerization. We studied this reaction over hydroxyapatites modified by substitution of the hydroxyl groups by fluoride. This notably enabled increasing the selectivity to acrylic acid while reducing the formation of the undesired acetaldehyde. Introduction of fluoride induced a modification of the phosphate (${\text{PO}}_{4}^{3-}$) groups. In the presence of water, fluoride prevented the formation of hydrogenophosphate species (${\text{HPO}}_{4}^{2-}$), which are well-known acid sites responsible for the formation of acetaldehyde by decarboxylation/decarbonylation. Further, we evidenced an important impact of fluoride substitution on crystallinity, specific surface area and on the surface Ca/P ratio. This latter is known to be a key parameter to control the acidity and the basicity of the hydroxyapatites. Using FT-IR spectroscopy with propyne as a probe molecule, we could show that lactic acid was concertedly adsorbed on basic and acid sites, which might be at the origin of the observed superior performances.

## Introduction

Dehydration of lactic acid remains a challenge because of the technical and chemical hurdles it represents (Bonnotte et al., 2018). While some progress has been achieved in terms of selectivity, especially with phosphate catalysts, the various mechanisms leading to the formation of undesired by-products such as acetaldehyde remain a subject of debate (Holmen, 1958). Generally, whether studying zeolites or phosphates as catalytic systems, it is admitted that, unlike conventional dehydration (catalyzed in the gas phase) often carried out on relatively acidic catalysts, dehydration of the lactic acid requires a very fine association between acidic and basic sites (acid-base pairs) (Holmen, 1958; Bonnotte et al., 2018).

Catalytic dehydration reaction of lactic acid to acrylic acid was thoroughly studied not only in the gas phase, but also in water under supercritical and subcritical conditions (Mok et al., 1989; Hatada et al., 2004, 2005; Aida et al., 2009) or even indirectly (acetoxylation of the lactic acid to *2*-acetoxypropionic acid which is then pyrolyzed to acrylic acid) (Beerthuis et al., 2015). However, whether in the gas or liquid phase, several parallel, and secondary reactions limit the selectivity to acrylic acid. Among them, decarboxylation or decarbonylation of lactic acid to acetaldehyde are particularly limiting. In addition, hydrogen from the decarboxylation reaction of lactic acid can induce side reactions such as reduction of lactic acid or acrylic acid to propionic acid. Formation of acetaldehyde is favored over the formation of acrylic acid with activation energies of 115 and 137 kJ.mol^−1^, respectively, according to the calculations of Wadley et al. (1997). The formation of acetaldehyde is often attributed to the presence of medium and strong acid sites on the catalyst (Katryniok et al., 2010). The other products (*2,3*-pentanedione, acetic acid, and sometimes lactide and hydroxyacetone) are generally formed in smaller amounts. As aforementioned, due to the parallel or secondary reactions, it is thus difficult to obtain high yields of acrylic acid, and the catalyst therefore plays a key role in the orientation of the selectivities. Several types of catalysts such as zeolites or basic oxides have been largely tested in the catalytic gas-phase dehydration of lactic acid (Bonnotte et al., 2018). However, while hydroxyapatites (HAPs) behave both acid and basic sites, they have not been so much studied in this reaction. HAPs are a class of solids apart from phosphates, because of their structure and versatility in composition. As a main constituent of bones and teeth, this material has also been studied for a long time in the field of medicine for several applications such as bone integration (Zhou and Lee, 2011), dental implants (Kurashina et al., 1984), or drugs (Zhang et al., 2015). It also quickly became a subject of study for their application in basic or bi-functional catalysis, in particular in the synthesis of heavy alcohols with the Guerbet reaction (Tsuchida et al., 2008), the Knoevenagel condensation reaction (Sebti et al., 2002), the Michael addition (Gruselle et al., 2011), or for dehydration (Lan and Zhang, 2015), oxidation (Zhao et al., 2013) or dehydrogenation reactions (Hara et al., 2003). HAPs have the general chemical formula Ca_5_(PO_4_)_3_OH, but are generally described by the Ca_10_(PO_4_)_6_(OH)_2_ formula, which actually represents two molecules contained in the crystalline pattern (crystalline symmetry, space group 6/m) (Charlton et al., 2006). The composition of the hydroxyapatites is extremely variable, because not only they are rarely stoichiometric (Ca/P = 1.67), but also because each constitutive element can be, to a certain extent, substituted without losing the crystalline structure (Ben Osman, 2014): Ca^2+^ ions can be replaced by mono and divalent cations such as Sr^2+^, Ba^+^, Pb^2+^, Mg^2+^, Zn^2+^, and Na^+^. ${\text{PO}}_{4}^{3-}$ groups may be substituted with ${\text{CO}}_{3}^{2-}$, ${\text{AsO}}_{4}^{3-}$, ${\text{SiO}}_{4}^{4-}$, ${\text{VO}}_{4}^{3-}$, ${\text{SO}}_{4}^{2-}$, and ${\text{HPO}}_{4}^{2-}$. Similarly, the OH^−^ anions can be replaced by halogenic anions: F^−^, Cl^−^, Br^−^, or I^−^. These compositional variations obviously bring a very strong impact on the acidic and basic properties of solids. They are mainly responsible for modifications of sites' density but a modification of their strength is also observed, though to a lesser extent.

Therefore, the main objective of this work was to study the influence of the OH^−^ ions substitution by F^−^ on the chemical, physical and catalytic properties of hydroxyapatites. The synthetized materials were characterized using different physical and chemical techniques and tested in the gas phase dehydration of lactic acid to acrylic acid.

## Experimental

### Materials

Several solids were synthesized. The protocols for the preparation and synthesis of hydroxyapatites are described in details in the next sub-section. The reagents used for each synthesis were calcium nitrate hexahydrate ([Ca(NO_3_)_2_·6H_2_O], Sigma Aldrich), diammonium phosphate ([(NH_4_)_2_HPO_4_], WVR), an aqueous solution of ammonia (28 wt.%, NH_4_OH, Sigma Aldrich). The various substituents used were: ammonium fluoride and chloride ([NH_4_F], [NH_4_Cl], Sigma Aldrich), zinc, potassium and calcium nitrates ([Zn(NO_3_)_2_, 6H_2_O], KNO_3_, NaNO_3_, Sigma Aldrich). Lactic acid, acrylic acid, acetaldehyde and propionic acid were purchased from Sigma Aldrich. All reactants were of analytical grade and used as received.

### Catalysts Preparation

Among the different possible synthetic routes, the co-precipitation method was used in the present study. Depending on the desired composition of the hydroxyapatite, various adaptations of pH, temperature, reagents concentrations and maturation times were used. Two aqueous solutions containing the main reagents: the first solution (between 300 mL and 1 L) containing diammonium phosphate (and optionally an anionic substituent) and the second solution containing calcium nitrate (and optionally a cationic substituent) were prepared. The first solution was heated to the given temperature (65°C ≤ *T* ≤ 80°C) under magnetic stirring (600 rpm), and the pH was adjusted at a value of 9 or 10 with a peristaltic pump for injecting the aqueous solution of ammonia. The second solution was then added drop wise to the first solution (which took between 45 min and 2 hours), using a dropping funnel. During this process, the pH was maintained at the initial value set by adding controlled volume of the aqueous solution of ammonia. The solution was then kept under stirring at a given temperature for a time ranging from 2 to 6 h. After maturation, the formed solid was recovered by filtration on a vacuum flask equipped with a Buchner filter. After evacuation of the solution, the wet solid was washed several times with two liters of hot distilled water. As-obtained solid was then placed in an oven at 100°C overnight. Finally, the solid was grounded before calcination between 400 and 600°C, in a muffle furnace under static air for 2 h.

### Characterization

ICP-OES analyzes were carried out on an AGILENT 700 ICP-OES within the REALCAT platform applied to high throughput screening.

The carbon content analyzes were carried out at the CNRS Central Analysis Service in Villeurbanne using a MITSUBISHI oxidizing combustion device coupled to DIONEX ion exchange chromatography.

The specific surface area (SA [m^2^.g^−1^]) of the catalysts was evaluated using adsorption/desorption of nitrogen on a MICROMERITICS ASAP 2000 analyzer after degassing during 3 h at 300°C. SA was determined using the Brunauer-Emett-Teller (BET) calculation method using the isotherm obtained at 77 K.

The crystalline phases were identified thanks to the X-Ray Diffraction (XRD) technique on a BRUKER AXS D8 Advance diffractometer configured in Bragg-Brentano geometry, equipped with a LynxEye Super linear detector and a CuKα X-ray source (λ = 1.5406 Å). The patterns were recorded at room temperature, with values of 2θ between 10 and 80°, and with a pitch of 0.02° and an acquisition time of 0.5 s. The lattice parameters were determined using the Le Bail method and JANA2006 Software (Petricek et al., 2014).

The surface composition (about 8 nm in depth) was determined using the X-ray Photoelectron Spectrometry (XPS) technique using a KRATOS ANALYTICAL Axis Ultra DLD spectrometer equipped with a monochromatic AlKα source (*h*υ = 1486.6 eV; 10 mA, 12 kV). The acquisition of the spectra was carried out with charge compensation, in a 10^−9^ mbar analysis chamber with a 40 eV passing energy for the high resolution and 160 eV for over-flights. The spectra were recalibrated using as a reference the C-C/C-H component of the C1s level set at 284.8 eV.

The acidity of the catalysts was evaluated using the ammonia-programmed temperature desorption (TPD-NH_3_) technique. The analysis was carried out thanks to a device allowing successive injection of calibrated loops of ammonia until saturation of the sample (*ca*. 50 mg) pretreated under helium at its initial calcination temperature (2 h, 10°C.min^−1^) in a quartz reactor. The adsorption was carried out between 40 and 130°C and desorption up to 600°C with a ramp of 10°C.min^−1^. The quantity of ammonia at the outlet of the reactor was evaluated by means of an ALPHAMOS gas chromatography (GC) equipped with a thermal conductivity detector (TCD).

The study of the adsorption of propyne followed by Fourier transform infrared transmission allowed us to study the acid and basic surface properties of our samples. Prior to analysis, these latter were finely ground and then pressed into self-supporting pellets using a hydraulic pressure press operating at 10^6^ Pascals. The as-obtained pellets were then transferred to the device described in Figure S1. After treatment at 400°C (samples' calcination temperature, 2 h, 5°C.min^−1^), the pellet was returned to room temperature and the cell was evacuated at a pressure of about 5.10^−2^ mbar. A reference spectrum was then recorded on a THERMO Nicolet 490 Fourier transform transmission infrared spectrometer between 400 and 4,000 cm^−1^ (resolution of 1 cm^−1^). Before lowering the pellet to the KBr window, a so-called “*background”* spectrum was acquired and subtracted from the recorded spectra. A volumetric volume section (2.124 cm^3^) enabled controlled pressure calibrated additions (~1.3–13 mbar) of propyne. When the pellet was saturated (spectrum shape no longer evolving), the cell was evacuated for 10 min and a last spectrum was recorded.

### Catalytic Tests

The catalytic dehydration of lactic acid was performed in a typical fixed bed reactor using the procedure described hereafter. The reactor was loaded with the catalyst and heated to the reaction temperature during the night before the test. The outlet lines were also heated to 190°C. The stabilization of the flow at the reactor inlet required at least 7 h; the “by-pass” section was placed under helium flow (between 15 and 30 mL.min^−1^), and the lactic acid solution was injected (between 20 and 50 wt.% in lactic acid, with a flow rate between 0.025 and 0.05 mL.min^−1^). Analysis of the products was carried out off-line, with a column adapted to all products resulting from the reaction: a semi-capillary column ZB-WAX-Plus (ZEBRON, PHENOMENEX) of 30 m, 0.53 mm external diameter and 1 μm film thickness (polyethylene glycol). The temperature and time of the reaction were fixed depending on the performed analysis.

## Results and Discussion

### Physical and Chemical Properties

In this study, 4 different hydroxyapatites were considered. In the case of the standard stoichiometric hydroxyapatite (Table 1, Entry 1) the final ICP Ca/P ratio is relatively close to the ratio imposed by the quantities of reagents used during the with a value of 1.62, thus slightly lower than the theoretical one of 1.67. In the case of fluorapatites, the theoretical fluorine substitution rates of 0.5, 1 and 2 correspond to fluorapatite Ca-XAP-F-OH (Table 1, Entry 2), Ca-XAP-F2 (Table 1, Entry 3) and Ca-XAP-F4 (Table 1, Entry 4), respectively. The Ca/P ratio increased with the increase of the F content in the materials and was equal or higher than the theoretical one.

**Table 1:** Catalytic materials.

**Entry**	Catalysts^*^	**Preparation conditions**	**ICP Ca/P ratio**	**XPS Ca/P ratio**	**BET SA (m^**2**^.g^**−1**^)**	**%F**
1	Ca-HAP-S	pH 10, *T* = 65°C	1.62	-	94.5	-
2	Ca-XAP-F-OH	pH 9, *T* = 65°C	1.66	1.50	79.4	1.60
3	Ca-XAP-F2	pH 9, *T* = 65°C	1.70	1.49	64.6	2.97
4	Ca-XAP-F4	pH 9, *T* = 65°C	1.73	1.54	53.7	4.15

* *Theoretical Ca/P ratio was 1.67 in all cases*.

Nitrogen adsorption/desorption isotherms of all the samples (not shown here) were of the IIb type (H3 type hysteresis) according to the IUPAC classification. This type of isotherm is characteristic of non-porous or macroporous materials. The SAs are listed in Table 1. Such relatively low SAs (<100 m^2^.g^−1^) are due to relatively low porosities of these materials because of the presence of rod aggregates or irregular grains that represent the most common morphologies observed for HAPs prepared by precipitation (Rodríguez-Lorenzo et al., 2003; Roche and Stanton, 2014). It is generally observed that SA tends to decrease with the increase of the fluorine substitution extent when the samples are calcined at 900°C (Rodríguez-Lorenzo et al., 2003; Roche and Stanton, 2014). In the present work a calcination temperature of 400°C was used and we cannot thus directly compare the values between both studies. However, they are consistent with other data reported in the literature even if the methods of preparation significantly differ (Matsuura et al., 2014; Silvester et al., 2014).

**Figure 1:**
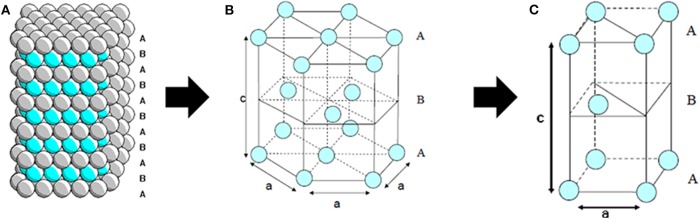
**(A)** Representation of ABABAB alternating layer stacks in a compact hexagonal arrangement. **(B)** Representation along the *c*-axis of a compact hexagonal structure. **(C)** Representation along the *c* axis of an elementary cell of a compact hexagonal system (Elliott et al., 2001).

We observed a clear—and progressive—SA drop, with a SA of 94.5 m^2^.g^−1^ between the reference hydroxyapatite (Table 1, Entry 1) and 53.7 m^2^.g^−1^ for fluorapatite containing the highest fluorine content (57%, Table 1, Entry 4). It is interesting to note that these observations go against those observed upon evolution of SA according to the Ca/P ratio (Tsuchida et al., 2009; Lamonier et al., 2011). Hydroxyapatites crystallize generally in a system very close to the hexagonal bipyramidal one (P63/m group) (Sudarsanan et al., 1972; Elliott et al., 2001). The fluorapatites can thus be represented in a compact hexagonal structure, alternating layers of PO_4_-tetrahedrals comparable to spheres of radius ~ 2.6 Å alternating in a ABABABAB feature as shown in Figure 1 (each blue or gray sphere represents an ${\text{PO}}_{4}^{3-}$ ion).

**Figure 2:**
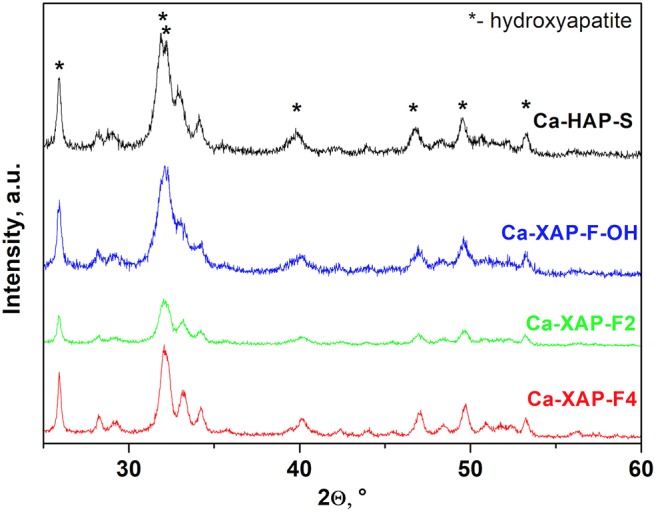
XRD patterns of the synthesized catalysts.

Depending on their size, ions like F^−^, Cl^−^, or OH^−^ occupy different positions along the *z*-axis passing through the center of triangles formed by Ca(II), or in the center of the triangle itself (*z* = 1/4 or *z* = 3/4) either when the size of the ion increases to an intermediate position between *z* = 1/4 and *z* = 1/2 or *z* = 3/4 and *z* = 1. The obtained diffractograms allowed to validate the formation of the apatite phase. The series of diffractograms (Figure 2) has well-defined peaks demonstrating the presence of a single apatite phase, and not an agglomeration of hydroxyl and fluorapatite phases. On the other hand, concerning the fluorapatite Ca-XAP-F4 (Figure 2) the XRD evidenced the presence of a mixture of fluorapatite and calcium fluoride (fluorite).

Moreover, the cell parameter values reported in Table 2 are consistent with those in the literature, particularly considering the work of Sudarsanan et al. who established that the cell parameters for a natural or synthetic fluorapatite are: *a* = 9.367 Å and *c* = 6.884 Å (Sudarsanan et al., 1972). These parameters are very close to those of Ca-XAP-F2 (although not all OH^−^ ions were substituted in that case). Moreover, the trend observed in Table 2 of a decrease in the *a* parameter is also in good agreement with the observation of other teams (Rodríguez-Lorenzo et al., 2003; Yao et al., 2009). Indeed, progressive substitution of OH^−^ ions by F^−^ ions causes a decrease in the *a* value directly correlated to the introduced amount of F^−^: as the phosphate atoms are not bonded to the Ca(I) atoms organized along the *z* axis, there will be no (or little) influence on the size of the cell in this direction. On the other hand, the F^−^ species located at the centers of the triangles formed by the Ca(II) ions will interact more strongly, by their electronegativity on these calcium ions than on the OH^−^ ions, inducing a “*compaction”* and thus a decrease in the size of the cell.

**Table 2:** Cell parameters determined from XRD.

**Catalyst**	***a* (A)**	***c* (A)**
Ca-HAP-S	9.417	6.884
Ca-HAP-F-OH	9.396	6.889
Ca-XAP-F_2_	9.368	6.889
Ca-XAP-F_4_	9.359	6.885

**Figure 3:**
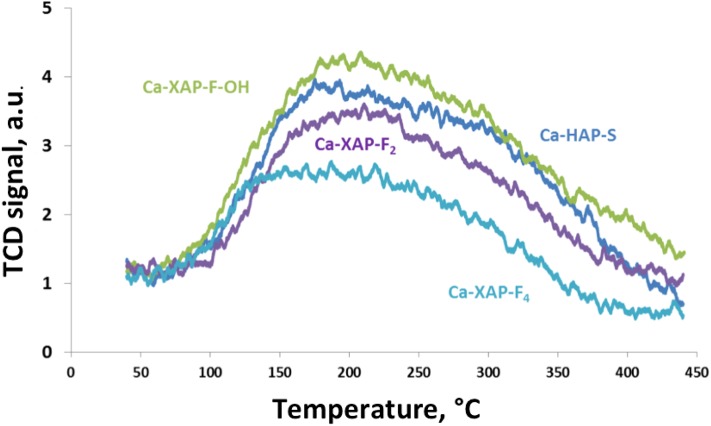
NH_3_ TPD profiles for Ca-HAP-S and F substituted hydroxyapatites.

**Figure 4:**
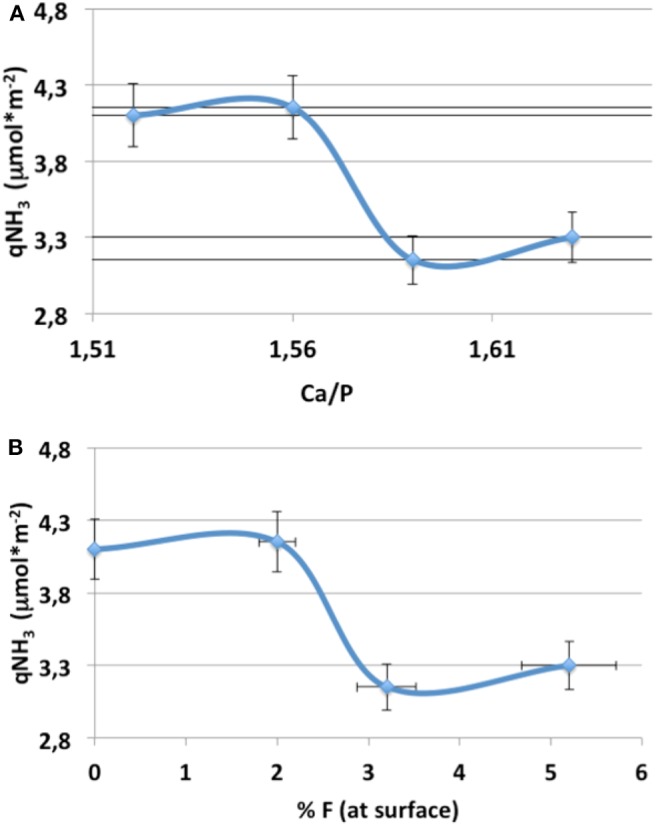
Quantity of ammonia desorbed per m^2^ of apatite **(A)** as a function of the Ca/P surface ratio and **(B)** as a function of the fluorine surface composition.

NH_3_ desorption profiles showed in Figure 3. They illustrate the decrease in the amount of acidic sites of fluorapatites with the gradual incorporation of fluorine. The total amount of NH_3_ desorbed clearly depends on the quantity of F incorporated to the catalyst (Table 3). The highest the amount of F, the lowest the quantity of desorbed NH_3_. This decrease can also be linked to the increase in the Ca/P ratio following the introduction of F. Indeed, it was observed very often in the literature that the acid/base properties of HAPs are highly, if not exclusively, depending on the Ca/P ratio (Ghantani et al., 2013; Bonnotte et al., 2018). It is quite obvious from Figure 4 that one cannot distinguish which element of composition actually induces this change in the acidic properties of the fluorapatites, as the Ca/P surface ratio is directly linked with the surface atomic percentage of F.

**Table 3:** Quantities of NH_3_ desorbed during the TPD-NH_3_ study.

**Catalyst**	NH_**3**_ [μmol*g^**−1**^]	NH_**3**_ [μmol*m^**2**^]
Ca-HAP-S	360	4.18
Ca-HAP-F-OH	394	4.20
Ca-XAP-F_2_	233	3.29
Ca-XAP-F_4_	198	3.65

In addition, since ammonia adsorbs indiscriminately on Lewis and Brønsted acid sites, the above experiment does not able to determine which population of these sites is actually involved. The main acid sites present in hydroxyapatites are believed to be calcium ions (Lewis acid sites). The Ca/P ratios in the studied catalysts present an over-stoichiometry, it can be assumed that, for reasons of balance of charges, the solid incorporates type B carbonates. The final formula of the fluorapatites would then be Ca_10−*x*_(PO_4_)_6−*x*_(CO_3_)_*x*_(OH)_2−y−0.5*x*_F_y−0.5*x*_, which can explain the increase in the Ca/P ratio with the substitution of ${\text{PO}}_{4}^{3-}$ groups by ${\text{CO}}_{3}^{2-}$ ions and the creation of OH^−^ and/or F^−^ vacancies. These latters may be considered as Lewis sites, as previously suggested by a work published by some of the present authors (Silvester et al., 2014). As the materials were synthetized under room atmosphere, it is therefore highly possible that carbonate ions are integrated into fluorapatites thanks to the dissolution of atmospheric CO_2_ in the basic solution. Thus, several acid sites can be distinguished:

– Ca^2+^: Although some authors identify calcium as a potential Lewis acid site such as Silvester (2013) and Silvester et al. (2014), who identified by them using XPS after adsorption of a probe molecule, *2*-phenylethylamine, other teams struggle to identify it by adsorption of other molecules such as CO (Diallo-Garcia et al., 2014a,b). More recently, the team of Hill et al. studied adsorption followed by DRIFTS of probes, with CO_2_ for basicity, pyridine and CO for acidity, and acetylene, and glycine for amphoteric properties on a stoichiometric hydroxyapatite. They showed the presence of very weak Lewis acid sites, and attributed this low Lewis acidity to Ca^2+^ species (Hill et al., 2015). Moreover, several authors demonstrated the adsorption of lactic acid on Ca^2+^ of HAPs, like authors specifically studying lactic acid dehydration to acrylic acid (Ghantani et al., 2013; Yan et al., 2014) or authors studying lactic acid adsorption on hydroxyapatites containing zinc (Turki et al., 2012);
– ${\text{HPO}}_{4}^{2-}$: These Brønsted acid sites are generated when the Ca/P ratio is over 1.67 [Ca_10−*x*_(HPO_4_)_*x*_(PO_4_)_6−*x*_(OH)_2−*x*_, *n*H_2_O]. In the presence of fluorine, the Ca/P ratio increasing, one would decrease the quantity of these sites, alongside with the quantity of vacancies [^δ+^];
– OH^−^/F^−^ [^δ+^] deficiencies: These deficiencies are also generated during under-stoichiometric calcium conditions and their presence is therefore linked to the presence of ${\text{HPO}}_{4}^{2-}$.

**Figure 5:**
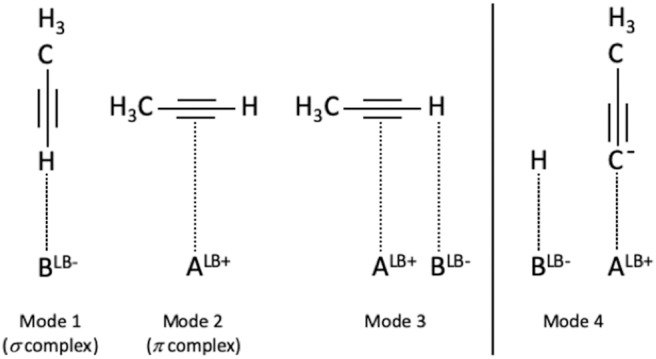
Non-dissociative (1 to 3) and dissociative (4) adsorption modes of propyne (Mordenti et al., 2001; Chizallet et al., 2006; Moulin et al., 2014).

The literature on propyne adsorption followed by infrared is relatively scarce. A few studies dealing with propyne (Thomasson et al., 1999; Hackler et al., 2001; Mordenti et al., 2001; Chizallet et al., 2006; Michalska et al., 2006; Valange et al., 2007; Moulin et al., 2014) or acetylene (Thomasson et al., 1999; Hill et al., 2015) adsorption on MgO, La_2_O_3_, ZrO_2_, CaHAP, CaO on MOF(X) (Metal-Organic Framework containing halogens). Propyne has a C≡ C triple bond able of interacting with an A^LB+^ (Lewis or Brønsted acid site) and a ≡C-H acetylenic proton able of interacting with a basic B^LB−^ site (Lewis or Brønsted basic site). This allows several adsorption modes, including a dissociative one. The various adsorption modes are summarized in Figure 5.

Figure S2 shows the gas phase propyne FTIR spectrum. It presents stretching bands of C≡C and ≡C-H bonds reported in Table S1. In addition, Figure S3 shows all the spectra obtained after the addition of 6.5 μmol of propyne as well as the spectra after evacuation under vacuum for 10 min. Each spectrum presented results from the subtraction between the measured spectrum and the reference spectrum of the pellet recorded before propyne addition. The presence of fairly intense bands in the υ_(≡C−H)_ region and low intensity bands in the υ_(C≡C)_ region can be observed. Such bands almost completely disappeared after evacuation. On the other hand, it is also worth to note the appearance of wide bands centered around 1,640 cm^−1^ and bands in the OH region around 3,700 cm^−1^ which are exalted after evacuation. These bands could be due to the presence of water adsorbed on the surface, as also evidenced by the shoulder in the 2,800–3,700 cm^−1^ range (Figure S3).

**Figure 6:**
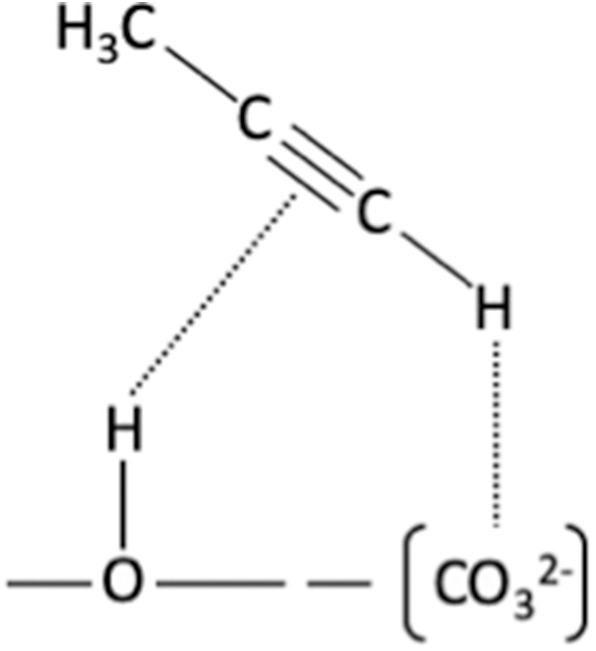
Adsorption scheme based on IR band allocations for propyne adsorption on lanthanum oxides (Valange et al., 2007).

**Figure 7:**
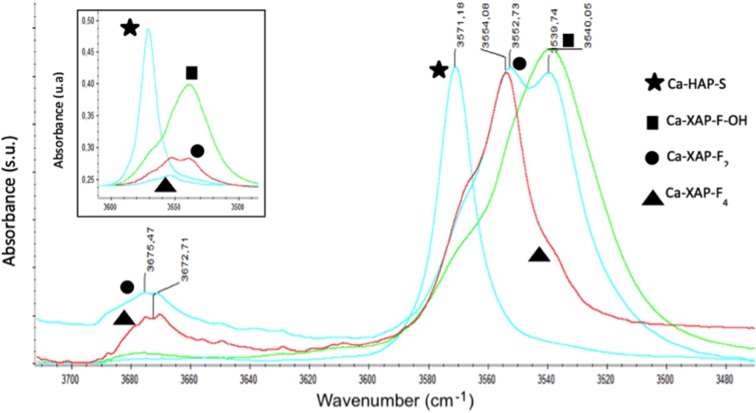
Absorbance IR spectra of the samples in the OH range.

A glance at Figure S4 and Table S3 suggests that the υ_(≡C−H)_ band is moving less and less strongly toward red with the presence of fluoride (“Δ” shift of −48 cm^−1^ for Ca-XAP-F4 to −54 cm^−1^ for Ca-XAP-F-OH). In addition, the intensity of the bands after evacuation decreases as the amount of fluorine increases, and the gas phase propyne band υ_(≡C−H)_ can also be observed for Ca-XAP-F2 and Ca-XAP-F4. However, we must take into account the smaller specific surface areas of these samples compared to Ca-HAP-S and Ca-XAP-F-OH. It should also be noted that the significant increase in the intensity of the band to 2,978 cm^−1^ attributed to υ_(−CH3)_ is difficult to explain. Two groups of samples can be distinguished in Figure S5 and corresponding Table S4: Ca-HAP-S and Ca-XAP-F-OH on one side, and Ca-XAP-F2 and Ca-XAP-F4 on the other side. The first group of samples mainly shows a band between 2,108 and 2,112 cm^−1^, which represents an average shift of −31 cm^−1^, which further increases in intensity. Again, under evacuation, the intensity of this band decreases sharply, suggesting a weak interaction with the surface. It is relatively difficult to decide on the value of the displacement of this band and its associated adsorption mode when referring to the literature. For example, Chizallet et al. who studied propyne adsorption on dehydroxylated and bare magnesium oxide surfaces (pellets treated at 750°C) observed a displacement of υ_(C≡C)_ of Δ = −50 cm^−1^ (Chizallet et al., 2006) while Huber *et al*. who also studied magnesium oxide surfaces (pellets treated at 750°C) observed a Δ shift of −19 cm^−1^ (Bailly et al., 2005). The first team assumed the presence of species adsorbed in associative mode 3 (Figure 5), but they mentioned that propyne is probably dissociated at these sites, based on the formation of a new band in the OH zone (3,441 cm^−1^), which is disturbed by additional increments in the amount of introduced propyne. On the other hand, the second team assigned the two observed bands at 2,123 cm^−1^ (Δ = −19 cm^−1^) and 2,094 cm^−1^ (Δ = −48 cm^−1^), respectively, to modes 1 and 2 (Figure 5). For mode 1, the ≡C-H bond is highly perturbed and the authors associate it with a band located at 3,280 cm^−1^, a band that is also observed in the present study. On the other hand, the shift observed in the C≡C zone being higher than that observed by Huber et al., it suggests a type 3 adsorption mode. In addition, the team of Valange et al. obtained results quite similar to ours, by studying the adsorption of propyne on lanthanum oxides with carbonates on their surface, like in our case. They propose the adsorption scheme of Figure 6 (Valange et al., 2007). To this type of adsorption (mode 3), they combine a υ_(≡C−H)_ band at 3,250 cm^−1^ (Δ = −84 cm^−1^) and a υ_(C≡C)_ band at 2,115 cm^−1^ (Δ = −27 cm^−1^). It was therefore assumed that this was also the main mode of adsorption of propyne present in this work. However, the presence of a shoulder at 2,082 cm^−1^ (in case of Ca-HAP-S) revealing a band as the amount of fluorine increases (2,086 cm^−1^ band for Ca-XAP-F4), suggests the generation of more acidic adsorption sites for the C≡C bond. Note also the presence of a low intensity band at 2,054 cm^−1^, close to that observed by Chizallet et al. at 2,045 cm^−1^ (Chizallet et al., 2006). The presence of these sites could lead to propyne dissociation, stabilized in propynide ion and OH^−^ ion (adsorption mode 4), as reported for magnesium oxide (Chizallet et al., 2006) or zinc oxide (Nakajima et al., 1982). Therefore, a close study of the OH^−^ zone in order to identify the possible presence of new bands, potentially resulting from the dissociative adsorption of propyne was carried out (Figure S6, Table S5). New bands appeared in the OH^−^ zone between 3,667 and 3,720 cm^−1^, thus in an area identified by Diallo-Garcia et al. as P-OH bands for hydroxyapatite (Diallo-Garcia et al., 2014a). These bands increased in intensity with the addition of propyne and the presence of fluorine in the material. The most intense and best-defined bands are thus visible for the Ca-XAP-F4 spectrum. As propyne additions progress, these bands showed a red shift until they are evacuated where they return to their starting position as when propyne was first added. These bands are also very intense and, unlike the bands attributed to propyne, they are at their maximum intensity after evacuation for each apatite. Finally, the other bands associated with OH^−^, as described in Figure 7, are not—or only slightly—affected by propyne successive additions.

It could therefore be assumed that propyne dissociation occurs with successive additions of propyne, resulting in the formation of different POH species on the apatite surface. It is also possible to highlight the disruption of the bands associated with phosphates, which implies them as at least one of the adsorption sites of propyne (Figure S7). However, the possible dissociation of propyne can be still a matter of debates, as it has never been reported on apatites so far. In addition, in similar studies with acetylene, this dissociation has not been observed (Diallo-Garcia et al., 2014b; Hill et al., 2015). Finally, there remains an area of the spectrum around 1650 cm^−1^ for which an evolution of the bands similar to that observed in the OH region is observed. The formation of these OHs would be linked to the formation of another adsorbed species, evidenced by a wide and intense band (Figure S8, Table S6). The bands in Figure S8 increase in intensity with successive additions of propyne before evacuation. During the addition of propyne, only the band centered around 1,635 to 1,645 cm^−1^ is visible. After evacuation, new bands appear only for Ca-HAP-S, at 1,697, 1,599, and 1,576 cm^−1^. It is difficult to interpret the origin of these bands because they do not correspond to any specific or expected bands. Two hypotheses can be formulated:

– Formation of carbonates or hydrogenocarbonates: In their work on the identification of acid-base pairs, Diallo-Garcia et al. studied CO_2_ adsorption followed by infrared and observed CO_2_ reactive adsorption (Diallo-Garcia et al., 2014a). This latter indeed reacts with the surface OH^−^ to form ${\text{CO}}_{3}^{2-}$ or ${\text{HCO}}_{3}^{-}$ ions and water according to the following reaction: CO_2_ + 2OH^−^ = ${\text{CO}}_{3}^{2-}$ + H_2_O. Note that some CO_2_ was identified in the present work in the gas phase propyne IR spectrum (see Figure S2 and Tables S1, S2). The formation of water could also explain the disturbance remaining after evacuation between 3800 and 2500 cm^−1^ as well as the intense and wide band centered around 1640 cm^−1^, as identified by Diallo-Garcia (2012) by FTIR with non-heat treated apatites. However, one can then wonder why the formation of water would be more important on Ca-XAP-F4, which has by far the most intense band around 1640 cm^−1^, whereas this fluorapatite is not supposed to contain OH^−^ or very little. The bands observed for Ca-HAP-S at 1697, 1599 and 1576 cm^−1^ are comparable to those identified by Diallo-Garcia et al. for hydrogencarbonates (Lauron-Pernot et al., 1991; Diallo-Garcia et al., 2011). The presence of water and carbonates is not impossible, but it is difficult to understand how their presence would increase when the presence of OH^−^ ions decreases. Nevertheless, one could think that the presence of water could be at the origin of these new bands in the OH area, as the evolution of the bands in the two respective regions seems similar.
– The formation of propargylic ions following propyne dissociation on acid-base pairs: It is very difficult to verify this hypothesis, as data on the formation of such species are scarce, and even non-existent on our materials. However, some data are available from Nakajima et al.'s work on butyne adsorption on ZnO (Nakajima et al., 1982). They attribute to propargylic species the bands located at 1,880 and 1,866 cm^−1^, which however remains far from our values. These species would gradually form during adsorption and some species would dissociate during evacuation. However, this hypothesis seems unlikely due to the observed very high intensities but especially due to the fact that apatites generally do not possess high strength acid or basic properties.

### Catalytic Tests

**Figure 8:**
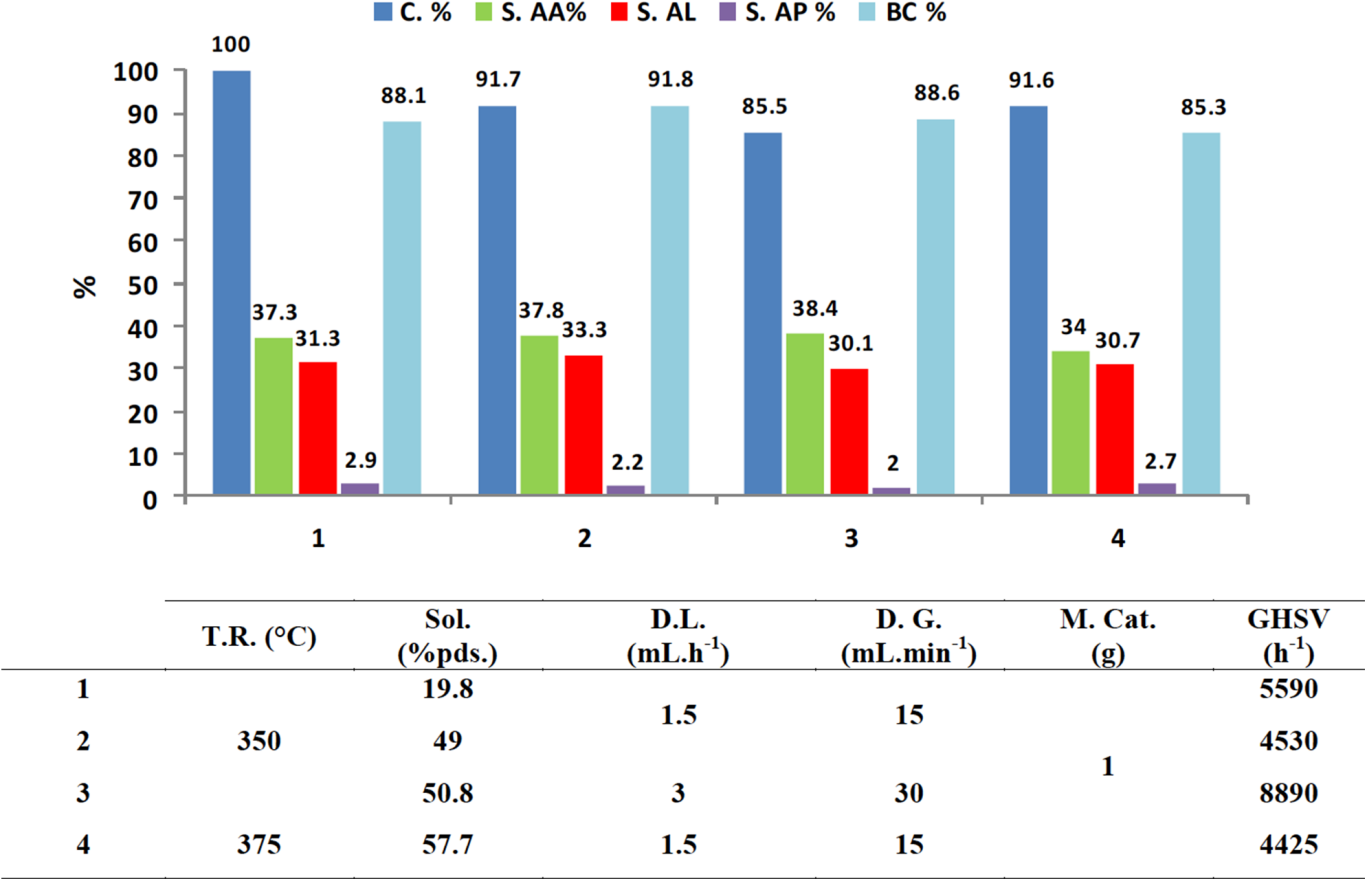
Catalytic activity in lactic acid dehydration for Ca-XAP-F2 catalyst under different experimental conditions. AA, acrylic acid; AL, acetaldehyde; AP, propionic acid; BC, carbon balance.

Concerning the catalytic properties, Ca-XAP-F2 catalyst was tested under various experimental conditions for sake of operating parameters optimization (Figure 8).

By varying the lactic acid concentration, gas and liquid flows, and reaction temperature, it can be seen that the selectivity and distribution of products is roughly unaffected. On the other hand, as expected, a slight decrease in conversion can be observed when the contact time decreases (increase in GHSV). However, while higher global performances could not be achieved, acrylic acid productivity increased. Indeed, when the lactic acid concentration increased by 250% or the GHSV by 200%, the acrylic acid yield decreased by 7% (from 37.3 to 34.7%) and 5.3% (from 34.7 to 32.82%), respectively.

**Figure 9:**
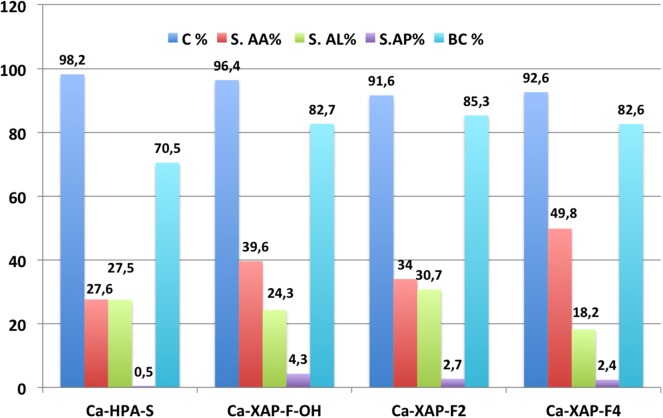
Catalytic activity in lactic acid dehydration for F substituted hydroxyapatites. AA, acrylic acid; AL, acetaldehyde; AP, propionic acid; BC, carbon balance; conditions: *T* = 375°C, GHSV = 4425 h^−1^, sol% = 57,5 wt.%, D.L = 1,5 ml*h^−2^.

In terms of performance, Ca-XAP-F2 catalyst stands out with a yield of acrylic acid of 37.3%, compared to 27.1% for the best performance obtained with HAP-S (Figure 9). A real effect of the fluoride ions on the catalytic performances is thus observed, with a total conversion and selectivity to acrylic acid significantly higher. Other fluorapatites were also tested. Results are given in Figure 9. It is difficult to draw clear trend from these catalytic tests' results (Figure 8) regarding the impact of the substitution extent of OH^−^ by F^−^ ions. Indeed, the largest acrylic acid yield (46.1%) is obtained with Ca-XAP-F4 while the minimum yield (31.1%) is obtained with Ca-XAP-F2. An intermediate value (38.2%) is obtained with Ca-XAP-FOH. The selectivity to acrylic acid is remarkably high, with a maximum of nearly 50% reached for Ca-XAP-F4.

**Figure 10:**
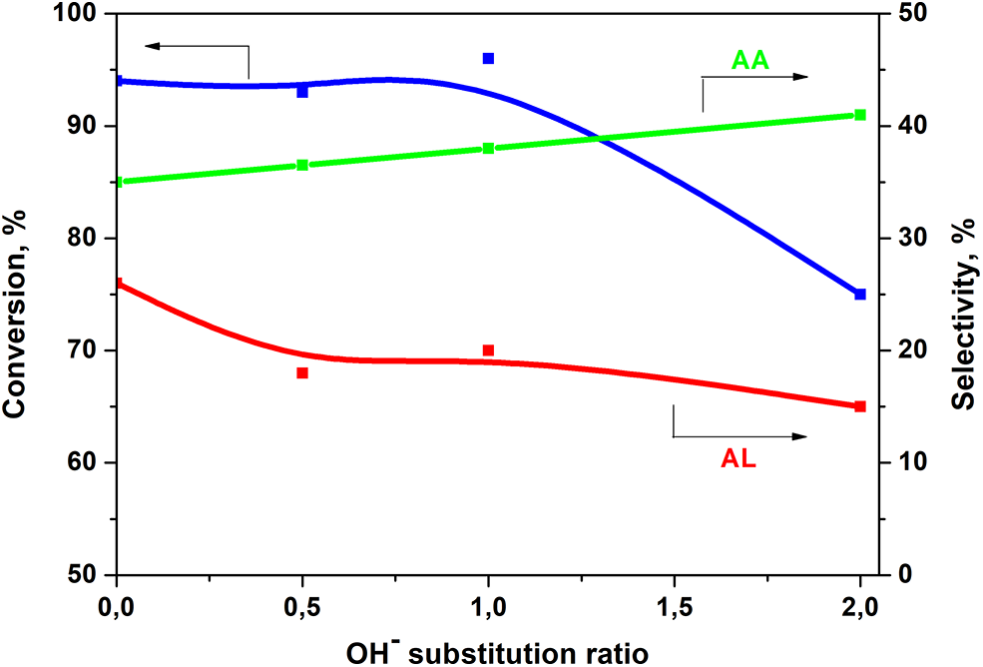
Selectivity to acrylic acid (AA) and acetaldehyde (AL) as a function of the OH^−^ substitution ratio.

It is quite difficult to explain why the yield drops in the presence of Ca-XAP-F2 compared to the other two fluorapatite compounds. One of the plausible explanations is the difference in catalyst preparation conditions. Indeed, Ca-XAP-F2 was prepared with different quantities and concentrations of precursors than the other Ca-XAPs, although the main parameters (molar ratios, reaction temperature, pH) are the same. Figure 10 presents the selectivities to acrylic acid and acetaldehyde obtained during the tests presented in Figure 9 as a function of the substitution ratio of OH^−^ by F^−^. The substitution ratio is 0 for Ca-HAP-S and 1 for Ca-XAP-F2.

An increase in acrylic acid selectivity, which can be directly correlated with that of the substitution rate of OH^−^ ions by F^−^ ions can be clearly seen in Figure 10. On the other hand, the changes in conversion and selectivity to acetaldehyde are similar, with a sharp drop above a substitution rate of 1 (from Ca-XAP-F2). There is therefore theoretically an optimal amount of fluorine that corresponds to a substitution rate between 1 and 2, beyond which the conversion of lactic acid falls. The best acrylic acid yield was obtained with Ca-XAP-F2 with more than 38%. On the other hand, the presence of fluoride ions not only improves selectivity to acrylic acid, but also reduces selectivity to acetaldehyde.

The formation of acetaldehyde in dehydration reaction in the gas phase is generally attributed to the presence of strong acid sites (Katryniok et al., 2010). This is also an important parameter in the conversion of lactic acid in the liquid phase under supercritical conditions, that are generally very acidic (Aida et al., 2009). In these conditions, lactic acid could be easily converted to acetaldehyde. It is difficult to obtain high selectivity to acrylic acid due to the various parallel or secondary reactions. Thus, the nature of the catalysts and their chemical compositions play a crucial role in the orientation of the reaction pathways. Several research group worked to understand the reaction mechanism. This could, without any doubt, enable further development of efficient catalysts and processes. Paparizos et al. studied aluminum phosphate catalyst for dehydration of lactic acid. They obtained an acrylic acid yield of 43.3% (Paparizos et al., 1988). Zhang et al. reported excellent catalytic performances with a mixture of potassium and barium phosphates catalysts (K:Ba = 40:60). They reported selectivities to acryclic acid of 92% with also a high conversion of lactic acid (91%) (Zhang et al., 2008). Matsuura et al. (2014) and Ghantani et al. (2013) studied also the dehydration of lactic acid over hydroxyapatites. Matsuura et al. obtained excellent yields (in the order of 70%) of acrylic acid depending on the Ca/P ratio. The authors concluded similarly to Blanco et al. (2014) that the acrylic acid formation depends on the balance between surface acid and base sites with a relatively moderate strengths and the absence of strong sites. On the other hand, Umbarkar et al. reported a total conversion of lactic acid associated with a 70% selectivity to acrylic acid (Ghantani et al., 2014). Very recently, the team of Yan et al. studied the effect of calcination temperature (from 360 to 700°C) on the catalytic performance of HAPs (Yan et al., 2014). They obtained the highest yield of acrylic acid of 62% at 360°C with a HAP (Ca/P: 1.62) calcined at 360°C.

Phosphates are clearly an interesting solution for the development of efficient catalysts for the gase phase dehydration of lactic acid. However, it appears relatively difficult to identify which are the most active phases and which active sites are involved in the given mechanisms of the dehydration reaction on these catalysts by comparison with the litterature, as the reaction conditions in the various works are actually quite different.

## Conclusions

During the study of the dehydration reaction of lactic acid, large variations in the catalytic performances of the different hydroxyapatites were observed. Although the emphasis is often placed on the Ca/P ratio as a paramount parameter for controlling the acidic and basic properties of hydroxyapatites, we have shown that substitution of OH^−^ by a halogen, fluorine, has a beneficial impact on the dehydration reaction. Indeed, a stoichiometric replacement of these OH^−^ ions by F^−^ ions not only improves the selectivity to acrylic acid but also significantly reduces the selectivity to acetaldehyde.

Compositional changes with respect to the non-substituted reference hydroxyapatite strongly impacted the specific surface area of the samples. Increase in the Ca/P ratio alone cannot explain the decrease in specific surface area upon F^−^ introduction, which should be attributed, at least in part, to the change in the density of the material, following a compaction of the triangles of Ca (II).

Acid-base properties of the samples were assessed by IR characterization of propyne adsorption. Some correlations could be observed between the presence of fluorine and the red shift of the propyne υ_(≡C−H)_ and υ_(C≡C)_ bands. It is possible that the presence of fluorine modifies both the distance between the acid site and the basic site (acid-base pair as adsorption site) while modifying the strength of the sites, in particular the potential acid site identified, as the Ca^2+^ cation.

Finally, the F^−^-substituted samples showed superior catalytic performances compared to the native non-substituted sample, which could be linked to the structural/strength modification of the surface acid-base pairs suggested by FTIR-followed propyne adsorption experiments.

## Data Availability Statement

The original contributions presented in the study are included in the article/Supplementary Material, further inquiries can be directed to the corresponding author/s.

## Author Contributions

All authors listed have made a substantial, direct and intellectual contribution to the work, and approved it for publication.

## Conflict of Interest

The authors declare that the research was conducted in the absence of any commercial or financial relationships that could be construed as a potential conflict of interest.
