# Supplementary material for: Lactic Acid Conversion to Acrylic Acid Over Fluoride-Substituted Hydroxyapatites
Source: Front Chem. 2020 May 13;8:421. doi: 10.3389/fchem.2020.00421 (PMC7237761; doi:10.3389/fchem.2020.00421)
Supplement: Supplementary file 1 [file Data_Sheet_1.docx]

Supplementary Material

# Lactic acid conversion to acrylic acid over fluoride-substituted hydroxyapatites

Robert Wojcieszak*, Thomas Bonnotte, Sébastien Paul, Benjamin Katryniok, Franck Dumeignil*

*Univ. Lille, CNRS, Centrale Lille, Univ. Artois, UMR 8181 - UCCS - Unité de Catalyse et Chimie du Solide, F-59000 Lille, France*

*Corresponding authors: [robert.wojcieszak@univ-lille.fr](mailto:robert.wojcieszak@univ-lille.fr); [franck.dumeignil@univ-lille.fr](mailto:franck.dumeignil@univ-lille.fr)


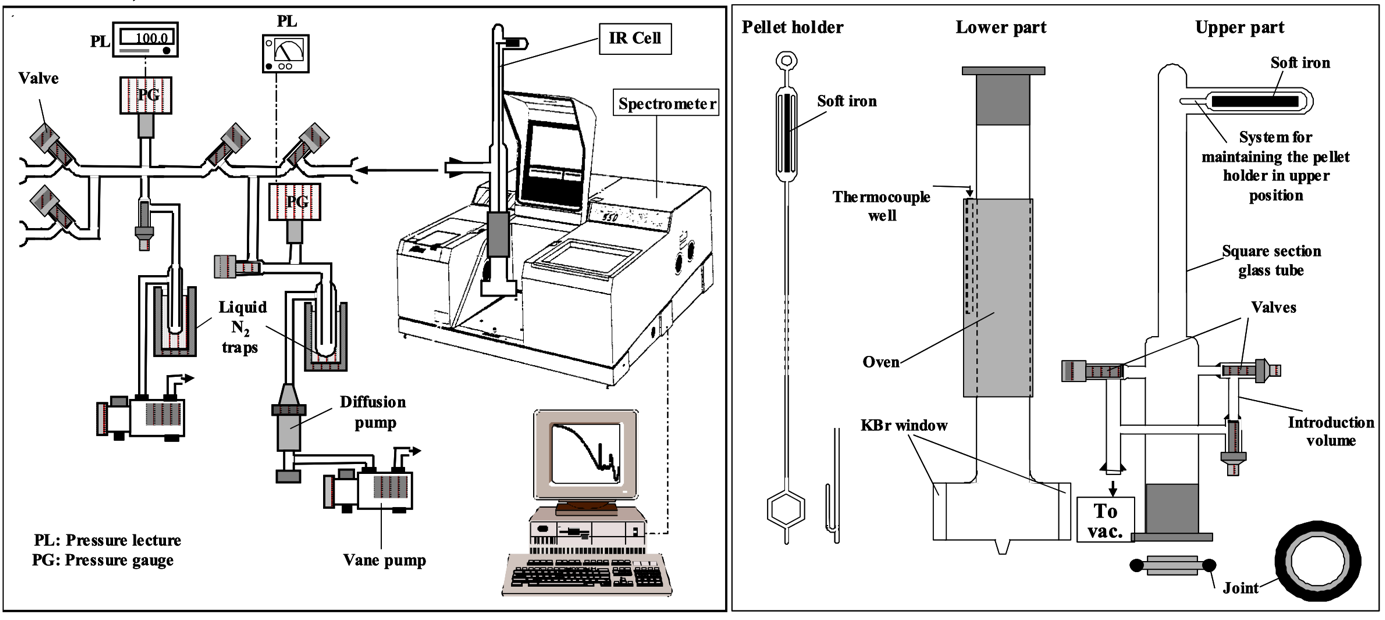


**Figure S1**. Schematics of the IR device/cell used for propyne adsorption experiments


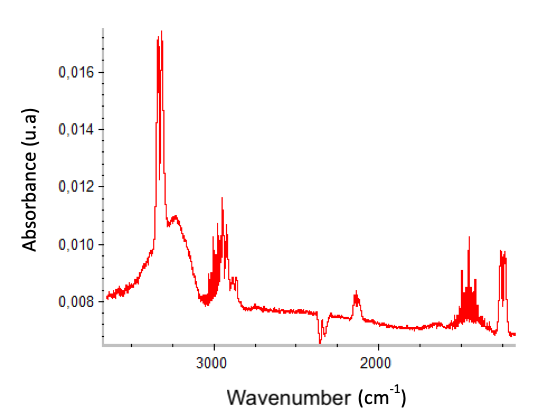


**Figure S2.** Room temperature transmission infra-red spectrum of gas phase propyne


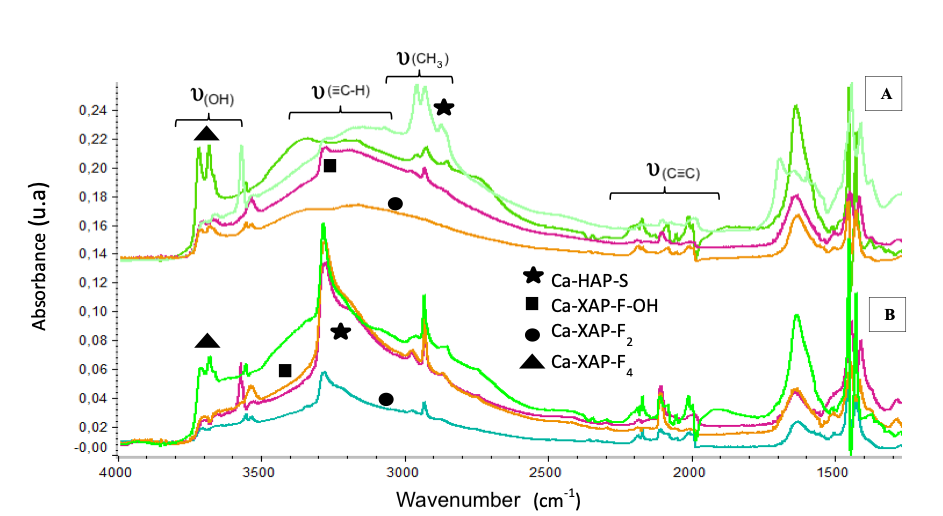


**Figure S3.** 1300-4000 cm^-1^ range of IR spectra of the samples after addition of 6.5 µmol of propyne (B) and after evacuation under vacuum for 10 min (A)


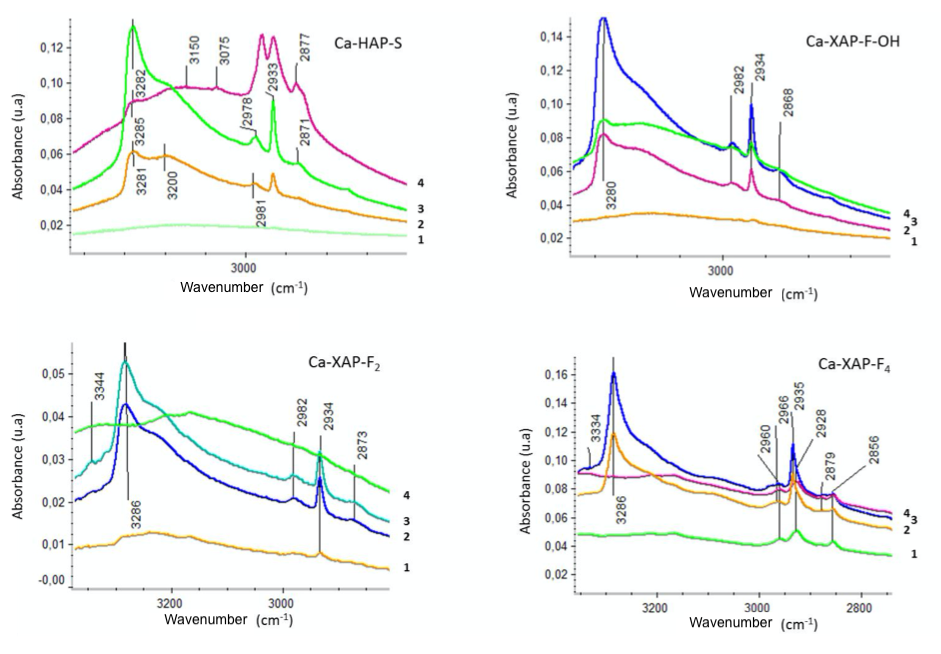


**Figure S4.** 1300-4000 cm^-1^ range of the IR spectra of the samples after calibrated additions of propyne (1 to 3) and after evacuation (4).


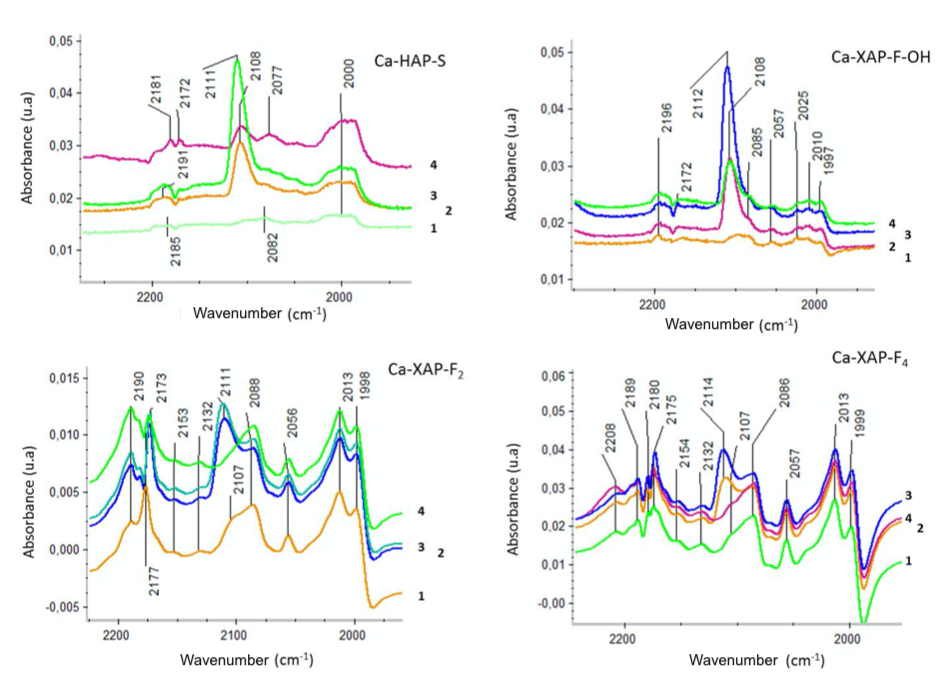


**Figure S5.** 2000-2200 cm^-1^ range of the IR spectra of the samples after calibrated additions of propyne (1 to 3) and after evacuation (4).


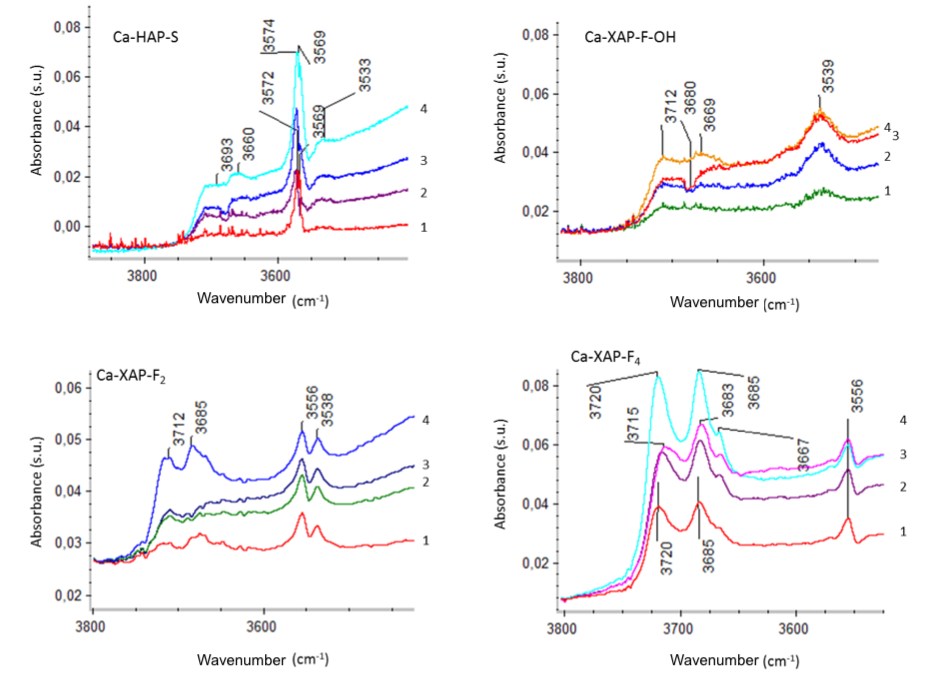


**Figure S6.** 3500-3800 cm^-1^ range of the IR spectra of the samples after calibrated additions of propyne (1 to 3) and after evacuation (4).


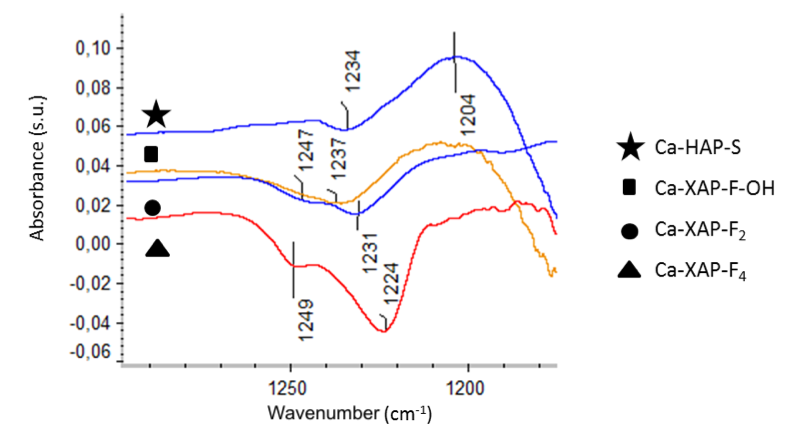


**Figure S7.** IR spectra of the samples in the 1200-1300 cm^-1^ range observed after evacuation, and showing perturbation of PO_4_^3-^ species.


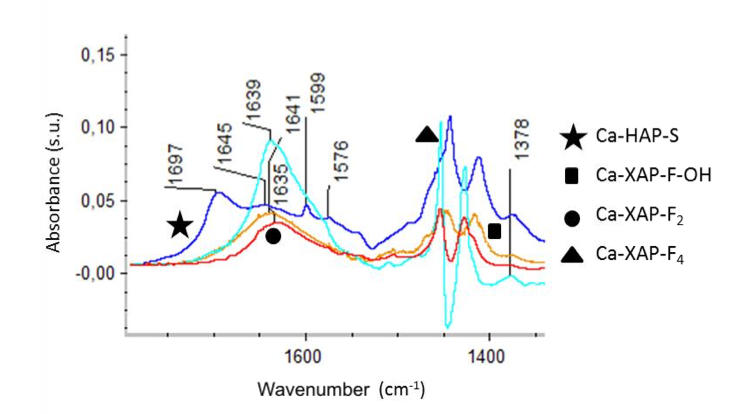


**Figure S8.** 1400-1750 cm^-1^ range of the IR spectra obtained after evacuation.

**Table S1.** Assignment of the IR bands observed on the gas phase spectrum of propyne.

| Wavenumber (cm^-1^) | Assignment | | Reference |
| --- | --- | --- | --- |
| 3334 | 3334 | υ_(≡C-H)_ | [1, 2] |
| 2141 | 2134 / 2141 | υ_(C≡C)_ | [1, 2] |
| 2349 | Asymmetric CO_2_ vibration? | | - |
| 3500-3071 | Water? | |  |

**Table S2.** Legend corresponding to the following figures for the study of the effect of calibrated additions of propyne.

| Total amount of added propyne (µmol) | | |  |
| --- | --- | --- | --- |
| 1 | 2 | 3 | 4 |
| 0.1 | 1.85 | 6.5 | evacuation |

**Table S3.** Remarkable bands in the 1300-4000 cm^-1^ region and their respective shifts (Δ). Units are cm^-1^_._

|  | Sample | | | | | | | | | Ref |
| --- | --- | --- | --- | --- | --- | --- | --- | --- | --- | --- |
|  | Ca-HAP-S | | Ca-XAP-F-OH | | | Ca-XAP-F_2_ | | Ca-XAP-F_4_ | |  |
| Spectrum number  3 | - | | 3443 | | | 3443 | | 3444 | | [2] |
|  |  |  | 3334 (gas phase) | | | 3333(gas phase) | | 3334 (gas phase) | | [1,2] |
|  | Ba 3281 |  | Ba 3280 | | Δ -54 | Ba 3286 | Δ -48 | Ba 3286 | Δ -48 | [3] |
|  | Sh 3222-3016 | Δ -134 | Sh 3232-3008 | | Δ -130 | Sh 3232-3008 | Δ -118 | Sh 3238-3016 | Δ -118 | [1-3] |
|  | 2978  2933  2877 | | 2982  2934  2968 | | | 2982  2934  2873 | | 2960 (1.4) - 2966(2.3)  2928 (1.4) / 2928+2934(2.3)  2856 | | 2922 ^[^2]  2861 [2] |
| Spectrum number 4 | 3282 | Δ -52 | 3280 | Δ -54 | | - (too low) | | 3288 | Δ -46 | 3250 [2]  3168 [2] |
|  | 3150 | Δ -184 | 3192 | Δ -142 | | 3192 | Δ -142 | 3210 | Δ -124 |  |
|  | 3075 | Δ -259 | 3170 | Δ -164 | | 3170 | Δ -164 | 3170 | Δ -161 |  |

Ba = Band position / Sh = shoulder position

**Table S4.** Remarkable bands in the 2000-2200 cm^-1^ region and their respective shifts (Δ).

|  | Sample | | | | | | | | Ref |
| --- | --- | --- | --- | --- | --- | --- | --- | --- | --- |
|  | Ca-HAP-S | | Ca-XAP-F-OH | | Ca-XAP-F_2_ | | Ca-XAP-F_4_ | |  |
| Bands (Ba) /  Shoulders (Sh)  (cm^-1^) | Ba 2111 (3)  Ba 2108 (2.4) | Δ -30  Δ -33 | Ba 2112 (3)  Ba 2108 (1.4) | Δ -29  Δ -33 | Ba 2111 (2.3)  Sh 2107 (1) | Δ -30  Δ -34 | Ba 2114 (2.3)  Sh 2107 (1.4) | Δ -27  Δ -34 | [1, 2] |
|  | Sh 2082 (1) | Δ -59 | Sh 2085 (2.3.4) | Δ -56 | Ba 2088 (1.2.3.4) | Δ -53 | Ba 2086  (1.2.3.4) | Δ -55 | [1, 4] |
|  | Ba 2077 (4) | Δ -64 | - | | | | | | [2] |
|  | - | | Ba 2057  (1.2.3.4) | Δ -84 | Ba 2054  (1.2.3.4) | Δ -87 | Ba 2057  (1.2.3.4) | Δ -84 | [5] |

**Table S5.** Remarkable bands in the 3500-3800 cm^-1^ region and their respective shifts (Δ).

|  | Sample | | | | | | | |
| --- | --- | --- | --- | --- | --- | --- | --- | --- |
|  | Ca-HAP-S | | Ca-XAP-F-OH | | Ca-XAP-F_2_ | | Ca-XAP-F_4_ | |
| Bands (Ba) /  Shoulders (Sh)  (cm^-1^) | Ba | 3693 | Ba | 3712 | Ba | 3712 | Ba | 3720/3715  3685/3683 |
|  |  | 3660 |  | 3669 |  | 3685 | Sh | 3667 |
|  | Ba | 3572/3574 | Ba | 3539 | Ba | 3556 | Ba | 3556 |
|  |  | 3569 |  |  |  | 3538 |  |  |

**Table S6.** Remarkable bands in the 1400-1750 cm^-1^ region.

|  | Apatites | | | | | | | | Ref |
| --- | --- | --- | --- | --- | --- | --- | --- | --- | --- |
|  | Ca-HAP-S | | Ca-XAP-F-OH | | Ca-XAP-F_2_ | | Ca-XAP-F_4_ | |  |
| Bands (Ba)  (cm^-1^) | Ba | 1697 | - | | | | | | [6] |
|  | Ba | 1645 | Ba | 1641 | Ba | 1635 | Ba | 1639 |  |
|  | Ba | 1599 | - | | | | | |  |
|  | Ba | 1576 |  |  |  |  |  |  |  |

References:

[1] Mordenti, D., Grotz, P., Knözinger, H., *Methylacetylene and tert-butylacetylene as IR-probe molecules for the characterisation of basicity,* Catalysis Today, 2001. **70**(1–3): p. 83-90.

[2] Chizallet, C., Bailly, M.L., Costentin, G., Lauron-Pernot, H., Krafft, J.-M., Bazin, P., Saussez, J., Che, M., Thermodynamic brønsted basicity of clean MgO surfaces determined by their deprotonation ability: Role of Mg2+–O2− pairs. Catalysis Today, 2006. **116**(2): p. 196-205.

[3] Thomasson, P., Tyagi, O.S., Knözinger, H., *Characterisation of the basicity of modified MgO-catalysts,* Applied Catalysis A: General, 1999. **181**(1): p. 181-188.

[4] Leclerc, H., Vimont, A., Lavalley, J.-C., Daturi, M., Wiersum, A.D., Llewellyn, P.L., Horcajada, P., Ferey, G., Serre, C., *Infrared study of the influence of reducible iron(III) metal sites on the adsorption of CO, CO2, propane, propene and propyne in the mesoporous metal-organic framework MIL-100,* Physical Chemistry Chemical Physics, 2011. **13**(24): p. 11748-11756.

[5] Valange, S., Beauchaud, A., Barrault, J., Gabelica, Z., Daturi, M., Can, F., *Lanthanum oxides for the selective synthesis of phytosterol esters: Correlation between catalytic and acid–base properties,* Journal of Catalysis, 2007. **251**(1): p. 113-122.

[6] Diallo-Garcia, S., Ben Osman, M., Krafft, J.-M., Boujday, S., Costentin, G., *Discrimination of infrared fingerprints of bulk and surface POH and OH of hydroxyapatites,* Catalysis Today, 2014. **226**(0): p. 81-88.
